# Microstructure and Hot Deformation Behavior of the Mg–8 wt.% Sn–1.5 wt.% Al Alloy

**DOI:** 10.3390/ma14082050

**Published:** 2021-04-19

**Authors:** Zhaoqian Sun, Yongjun Li, Kui Zhang, Xinggang Li, Minglong Ma, Guoliang Shi, Jiawei Yuan, Hongju Zhang

**Affiliations:** 1 State Key Laboratory of Nonferrous Metals and Processes, GRINM Co., Ltd., Beijing 100088, China; sunzhaoqiaq@163.com (Z.S.); zhkui@grinm.com (K.Z.); lixinggang@grinm.com (X.L.); maminglong@grinm.com (M.M.); shiguoliang@grinm.com (G.S.); yuanjiawei@grinm.com (J.Y.); 2 GRIMAT Engineering Institute Co., Ltd., Beijing 101407, China; 3 General Research Institute for Nonferrous Metals, Beijing 100088, China; 4 China United Test & Certification Co., Ltd., Beijing 101407, China; zhanghongju@gbtcgroup.com

**Keywords:** Mg–8 wt.% Sn–1.5 wt.% Al, hot deformation behavior, microstructure, processing map

## Abstract

Mg–Sn–Al alloy is a new type of heat-resistant magnesium alloy with great potential and the hot deformation process of this alloy is of great significance for its application. The microstructure, hot deformation behavior, textural evolution, and processing map of a Mg–8 wt.% Sn–1.5 wt.% Al alloy were studied. A Gleeble 1500 D thermo-mechanical simulator was used. The temperature of deformation was 653 to 773 K, the strain rate was 0.001–1 s^−1^, and the maximum deformation degree was 60%. The obtained results show that the rheological stress of the alloy decreases with an increase in deformation temperature and increases with an increase in the strain rate. The alloy is completely dynamically recrystallized at 653 K, and the entire structure is formed of homogeneous crystals/grains, with small secondary phase particles distributed at the crystal boundary. The mean apparent activation energy of hot compression deformation is 153.5 kJ/mol. The Mg–8 wt.% Sn–1.5 wt.% Al alloy exhibits excellent plastic deformation properties, an expansive thermal processing interval, and a narrow instability zone under the test temperature and deformation rate. The optimal process parameters of the alloy comprise deformation temperatures between 603 and 633 K and strain rates of 0.03 to 0.005 s^−1^.

## 1. Introduction

Heat-resistant magnesium alloys have attracted considerable attention as promising materials in the fields of aviation, automobile, and electronics [1]. They have the advantages of low density, high specific strength, high electromagnetic shielding, and easy machining [2,3]. Currently, the most commonly used heat-resistant magnesium alloys are Mg–RE magnesium alloys [4]; however, the price of rare earth (RE) metals is too high; thus [5], other types of magnesium alloys are also researched [6]. The Mg–Al and Mg–Zn alloys are also used as heat-resistant magnesium alloys, by adding rare-earth or alkaline-earth elements. Mg–Al alloys are low cost with good plastic deformation and Mg–Zn alloys have good heat conduction; however, the eutectic points of the Mg–Al and Mg–Zn alloys are 710 K and 614 K, respectively; so, the main second phase Mg_17_Al_12_ and MgZn_2_ show poor heat resistance properties, which prevents their use at higher temperatures [7,8].

Tin is a commonly used metal in industry, and it has a relatively low price [9]. Tin has large solid solubility in magnesium matrix, with the maximum of 14.62 wt.%, and it considerably decreases with a decrease in temperature [10]; this behavior is similar to that of rare-earth metals [11]. After dissolving in magnesium matrix, the second phase formed in the Mg–Sn alloy is the Mg_2_Sn phase [12]. It has a high melting point of 1044 K, high hardness, and it is still stable at a high temperature, which is similar to the behavior of Mg_5_RE [13]. Owing to similarity to Mg–RE magnesium alloys, Mg–Sn magnesium alloys have a great potential to be used as heat-resistant magnesium alloys [14,15]. Mg–Sn binary alloys are rarely used in industry due to poor mechanical properties [16], so aluminum was added as the third element to improve it; this mainly plays the role of solid solution strengthening [17]. Elsayed F R et al. [18] investigated the microstructure and thermal properties of Mg–Sn–Al alloy, and found that tin could inhibit the precipitation of Mg_17_Al_12_ phase, and due to the presence of Mg_2_Sn phase at the grain boundaries, the thermal properties and creep properties of the alloy are obviously improved; Kang D H, et al. [19] studied the ultimate tensile strength (UTS) and hot behavior of the Mg–8Sn–3Al–Si (wt.%) alloy, and it was found that the creep properties of the die casting alloy is better than that of AZ91; Liu J L et al. [20] studied the UTS of Mg-x Al-Y Sn (x = 1~9 wt.%, y = 1~3 wt.%) magnesium alloys, and it was found that the best performance that could be achieved is 260 MPa, with an elongation of 30%. The previous researchers studied the microstructure, mechanical properties, and creep properties of the Mg–Sn–Al alloys but few studies have been conducted on its hot deformation. Therefore, this study is mainly focused on the hot deformation behaviors of Mg–8Sn–1.5Al alloy. In practical applications, under deformation, magnesium alloys have good mechanical properties. However, magnesium alloy has a close-packed hexagonal structure; it has few slip systems, and it is difficult for it to undergo plastic deformation at room temperature; thus, magnesium alloys are always subjected to thermoplastic deformation [21]. Therefore, to objectively evaluate the heat resistance of the alloy, it is necessary to first conduct thermoplastic deformation of the alloy [22]. To obtain a reasonable regime, the alloy deformation process needs to be simulated before actual deformation. In this study, the hot deformation behavior of a Mg–8 wt.% Sn–1.5 wt.% Al alloy was researched.

## 2. Materials and Methods

A Mg–8 wt.% Sn–1.5 wt.% Al ingot was cast by using magnesium (99.95 wt.%), tin (99.98 wt.%), and aluminium (99.95 wt.%). Ar_2_ was used as the protective gas, as well as the tetrafluoroethane used as the cooling gas, and the ratio of them was 10:1. The crucibles were first preheated to 300 °C before the metals were added. Then, the ingots (9.05 kg Mg, 0.8 kg Sn and 0.15 kg Al) were added in turn and heated at 750 °C, insulated for 30 min and stirred until the metals had completely melted. Finally, the melt was cooled in water.

Homogenization heat treatment (480 °C, 24 h) was performed in a chamber furnace (SX–G30103). For the compression test, to achieve height reduction of up to 60%, samples with 10 mm diameter and 15 mm height were placed on the gleeble–1500 D thermal analog testing machine (Data Sciences International, Inc., St. Paul, MN, USA); the heating rate was 10 K/s, and the insulation time was 60 s. The experimental temperatures were 653, 693, 733, and 773 K, and the strain rates were 0.001, 0.01, 0.1, and 1 s^−1^. Sandpapers (600#, 1000#, 2000#, 5000#) were used to prepare the metallographic samples; after that, the samples were polished on the metallographic polishing machine (YMP-2) (Shanghai Optical Instrument Factory, Shanghai, China) for 35 s, with diamond polishing paste (1.0) coated on a polishing cloth. Electron backscattered diffraction (EBSD) samples were electrolytically polished (20 V, 15 s) on the basis of mechanical polishing, and nitric acid (20%) alcohol solution was used for that. The metallographic microstructures were obtained by optical microscopy (Carl Zeiss 2000 MAT) (Carl Zeiss Jena, Jena, Thuringia, Germany), and the grain boundaries and textures were observed by scanning electron microscopy (SEM, 7900 F) (Nippon Electronics Corporation, zhaoshima, Tokyo, Japan) instrument equipped with an EBSD system.

## 3. Results

### 3.1. Stress–Strain Curve

**Figure 1:**
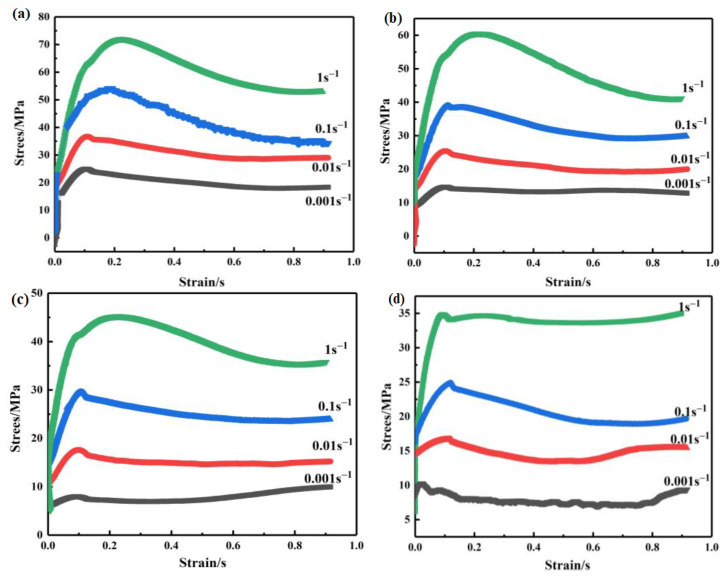
True stress–strain curves of the alloy during hot compression. (**a**) 653 K; (**b**) 693 K; (**c**) 733 K; (**d**) 773 K.

The stress–strain curves of the samples are shown in Figure 1. The abovementioned diagram shows that the stress rapidly increases with an increase in strain, then slowly increases to a peak value, followed by a decrease to a steady value, under the condition that the temperature and strain rate remain constant. Peak stress is usually an important factor in the ability of the alloy to be deformed. Therefore, peak stress was chosen as the representative to study the hot deformation behavior of the alloy, as shown in Table 1.

### 3.2. Constitutive Equation

It can be found that the peak stress varies with the temperature and the deformation strain rate (Figure 1). The plastic flow characteristics could be described by the constitutive relationship in metals and alloys, which contains material constants obtained from stress–strain data. It is particularly important for understanding hot deformation behaviors.

Zener and Sellars [23,24] use the modified Arrhenius formula to express the constitutive equation, which contains deformation activation energy (*Q*), temperature (*T*), and Zener–Hollomo parameters:

(1) $$Z=\exp\left(\frac{Q}{RT}\right)$$

where *Q* is the deformation activation energy, *R* is the gas constant (8.314 J/K), and *T* is the absolute temperature (in K).

The abovementioned relationship can be expressed by the exponential, power exponential, and hyperbolic sinusoidal function models [25]. For the low-stress state, the exponential function model (Equation (2)) is suitable; for the high-stress state, the power exponential function model (Equation (3)) is applicable. At the low-stress level activation energy:

(2) $$\overset{\cdot }{\varepsilon }=A_{1}\sigma^{n_{1}}\;\;(\alpha \sigma <0.8)$$

At the high-stress level:

(3) $$\overset{\cdot }{\varepsilon }=A_{2}\exp\left(\beta \sigma \right)\;\;\;(0.8<\alpha \sigma <1.2)$$

Correct once again:

(4) $$\overset{\cdot }{\varepsilon }=A[sinh\sigma \beta {]}^{n}\exp(\frac{-Q}{Rt})$$

where *A*_1_, *A*_2_, *n*_1_, *α,* and *β* are the material constants, which can be represented by the following relationship:

(5) $$\alpha =\beta /n_{1}$$

wherein, *α* and *n*_1_ can be obtained from Equations (2) and (3). Taking the natural logarithm of Equations (2) and (3), respectively:

(6) $$\ln\overset{\cdot }{\varepsilon }=ln+ln\sigma$$

(7) $$\ln\overset{\cdot }{\varepsilon }=lnA_{2}+\beta \sigma$$

**Figure 2:**
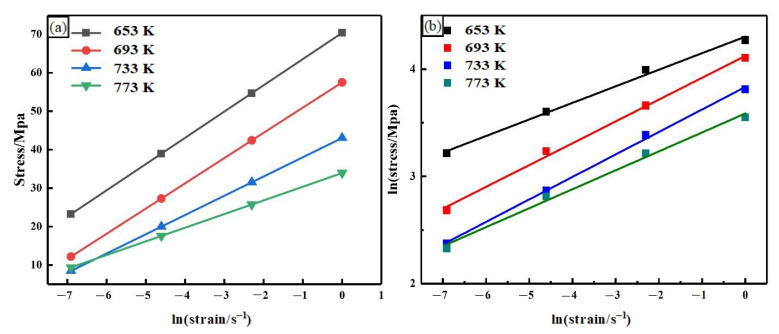
Fitting curve images of the peak stress and the strain rate. (**a**) Stress-ln(strain/s^−1^); (**b**) Ln(stress/MPa)-ln(strain/s-1).

*n*_1_ and *β* values can be obtained from Figure 2, and the results are shown in Table 2.

Taking the natural logarithm of Equation (4):

(8) $$\ln\overset{\cdot }{\varepsilon }=lnA-\frac{Q}{RT}+nln\left[sinh\left(\alpha \sigma \right)\right]$$

Then, taking the temperature as the independent variable to obtain partial differential:

(9) $$Q=R{\left[\frac{\partial ln\overset{\cdot }{\varepsilon }}{\partial ln\left(sinh\left(\alpha \sigma \right)\right)}\right]}_{T}{\left[\frac{\partial ln\left(sinh\left(\alpha \sigma \right)\right)}{\partial \left(1/T\right)}\right]}_{\overset{\cdot }{\varepsilon }}$$

**Figure 3:**
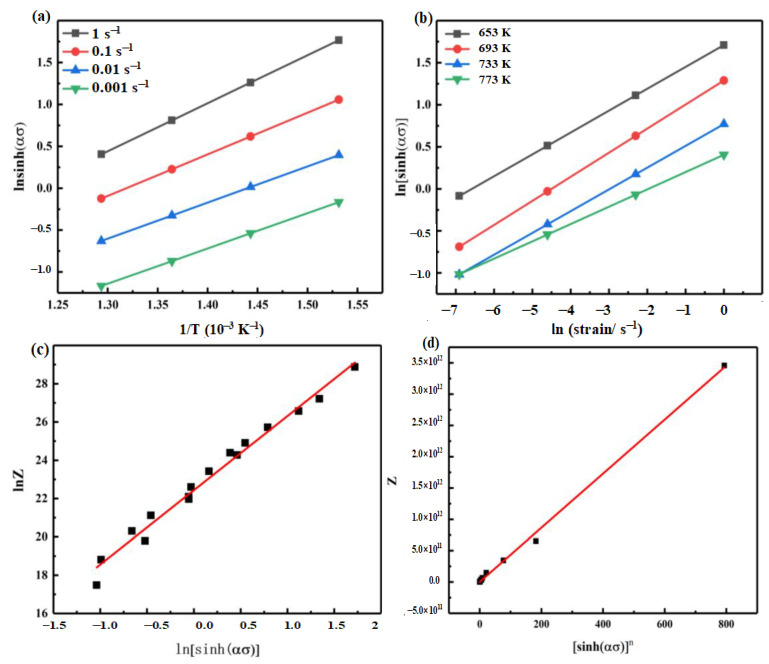
Fitting curve images in the calculation of the constitutive equation. (**a**) $\ln\left[\sinh\left(\alpha \sigma \right)\right]-1/T\left(10^{-3}{\;K}^{-1}\right)$; (**b**) $\ln\left[\sinh\left(\alpha \sigma \right)\right]-\ln\left(\mathrm{strain}/s^{-1}\right)$; (**c**) $\mathrm{lnZ}-\ln[\sin h\left(\alpha \sigma \right){]}^{n}$; (**d**)$\;Z-[\sin h\left(\alpha \sigma \right){]}^{n}$.

The two partial differentials in Equation (9) can be obtained according to Figure 3a,b. The slope of the curve slightly differs at different temperatures and strain rates; thus, the calculated value of activation energy is different at different conditions. According to the experimental results, the deformation activation energy of different temperatures and deformation strain rates was calculated; the average is 153.5 kJ/mol.

Substitute α into Equation (8), and draw $\mathrm{lnZ}-\ln[\sin h\left(\alpha \sigma \right){]}^{n}$ and $Z-[\sin h\left(\alpha \sigma \right){]}^{n}$, as shown in Figure 3c,d. The relationship between peak stress and strain rate during hot compression is obtained by linear regression, and it conforms well. After fitting, *N* is 3.88, *A* is 4.32 × 10^9^.

Constitutive equation of the alloy is:

(10) $$\overset{\cdot }{\varepsilon }=4.32\times 10^{9}{\left[sinh\left(0.034\sigma \right)\right]}^{3.88}\exp\left(\frac{-153,500}{RT}\right)$$

Calculation using the constitutive equation shows that the deformation activation energy (*Q* = 153.5 kJ/mol) and strain index (*n* = 3.88) of the alloy are obtained, which can verify and predict the machining properties of the alloy.

### 3.3. Processing Map

Based on the dynamic material model (DMM), the technique of metal processing mapping has been previously applied to the study of the hot deformation mechanism of magnesium alloys [26,27,28]. According to the DMM theory, the energy (*P*) absorbed per unit volume of the alloy during hot deformation is composed of two parts; one is heat dissipation (*G*), and the other is energy dissipation (*J*) related to the microstructure change during deformation. The relationship between them, the flow stress, and the strain rate can be expressed as follows:

(11) P=σ−G+J

The ratio of *J* to the linear dissipation is the power dissipation factor, expressed by *η*, as shown in Equation (12). In general, the greater the power dissipation factor *η*, the better the processing property of the material.

(12) $$\eta =\frac{J}{J_{max}}=\frac{2m}{m+1}$$

where *m* is the strain rate-sensitive coefficient of flow stress, and it can be obtained from $\left(\partial \mathrm{lg}\sigma \right)/(\partial \mathrm{lg}\;\overset{\cdot }{\varepsilon }$).

Prasad et al. [29] have used the principle of irreversible thermodynamic extremum to establish the instability criterion of the material during large plastic deformation and determined that it was unsteady rheology when the instability parameter could be obtained. In addition, the extreme principle of thermodynamics of irreversible processes is applied to a large plastic flow continuum, and a criterion for the occurrence of rheological instability is obtained. Its expression is as follows:

(13) $$\xi \left(\overset{\cdot }{\varepsilon }\right)=\frac{\partial lg\left[m/\left(m+1\right)\right]}{\partial lg\overset{\cdot }{\varepsilon }}+m\leq 0$$

**Figure 4:**
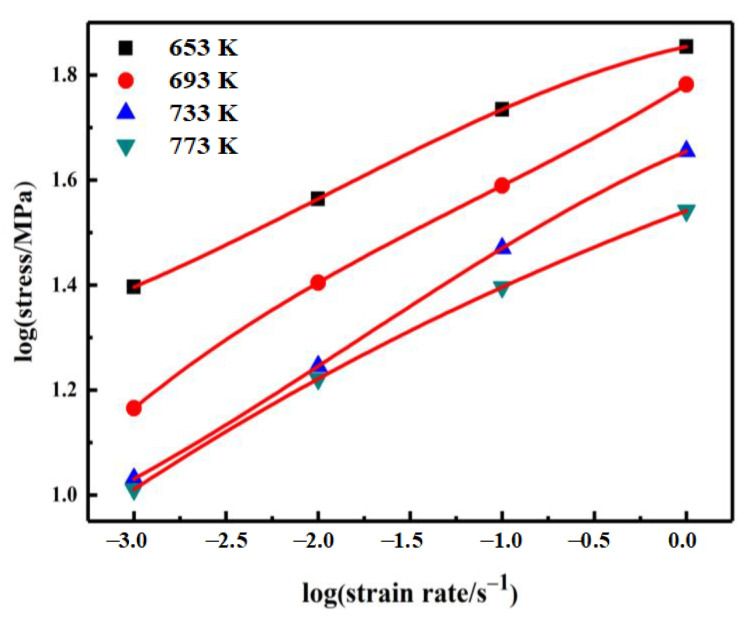
Interpolation curve of the flow stress and strain rate.

The relationship between ($\partial lg\sigma )\;and\;\left(\partial lg\overset{\cdot }{\varepsilon }\right)$ can be fitted by cubic spline functions, and the results are shown in Figure 4.

(14) $$\sigma =a+\mathrm{blg}\;\overset{\cdot }{\varepsilon }+\mathrm{clg}{\left(\overset{\cdot }{\varepsilon }\right)}^{2}+\mathrm{dlg}{\left(\overset{\cdot }{\varepsilon }\right)}^{3}$$

The strain rate-sensitive exponent (*m*) can be expressed as:

(15) $$m=\frac{\partial lg\sigma }{\partial lg\overset{\cdot }{\varepsilon }}=b+2c\left(lg\overset{\cdot }{\varepsilon }\right)+3d{\left(lg\overset{\cdot }{\varepsilon }\right)}^{2}$$

where *a*, *b*, *c*, and *d* are all temperature-dependent constants.

**Figure 5:**
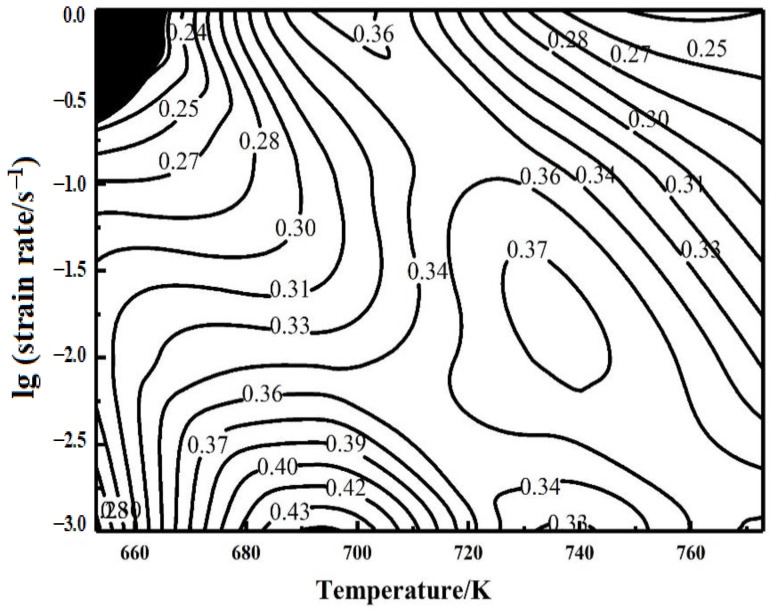
Processing map of the alloy.

After calculating *η*, the processing map of the Mg–8 wt.% Sn–1.5 wt.% Al alloy is drawn, and the results are shown in Figure 5. Outside the instability zone in the map, the higher the *η* value, the better the performance of the material. The instability area of the processing map is small at 653 K with the strain rate of 1 s^−1^, which indicates that the hot deformation of the alloy is excellent. The maximum dissipative power factor (*η*) of 0.43 appeared at 693 K, with the strain rate of 0.002 s^−1^, and the second is 0.37, which appeared at 733 K, with the strain rate of 0.01 s^−1^, which are all the suitable parameters for hot deformation. The minimum *η* is 0.24, which appears near the instability zone, and the second minimum η is 0.25, which appears at 773 K, 1 s^−1^; neither 653 K nor 773 K is the appropriate temperature for hot deformation.

## 4. Discussion

### 4.1. Effect of Temperature on Microstructure

**Figure 6:**
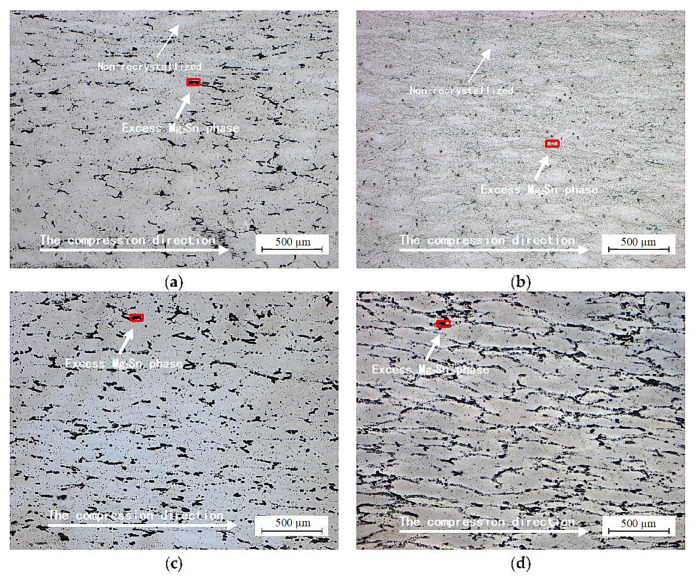
Microstructures of the alloy during hot compression at the strain rate of 0.001 s^−1^. (**a**) 653 K; (**b**) 693 K; (**c**) 733 K; (**d**) 773 K.

Temperature has a great effect on the hot deformation of the alloy. When the temperature is lower, dynamic recrystallization cannot fully complete; thus, refined crystalline strengthening of the alloy is difficult to achieve (Figure 6a). When the temperature is too high, with an increase in time, recrystallization grains begin to grow after dynamic recrystallization is completed (Figure 6d). Therefore, the control of temperature is crucial. Owing to its low stacking fault energy, it is difficult for dislocation in magnesium alloys to cross slip and climb during deformation; thus, dynamic recovery is difficult to achieve, and the dislocation density of the substructure is high, which promotes the nucleation of dynamic recrystallization and makes dynamic recrystallization easily occur [30]. When the deformation amount reaches a certain degree, dynamic recrystallization begins to occur (Figure 6b), and then complete dynamic recrystallization occurs (Figure 6c) and stress reaches a stable value. However, if the deformation temperature is too high, secondary dynamic recrystallization will occur, and some of the grains start to increase in size, and gradually absorb nearby grains to form larger grains. When the temperature is sufficiently high, the secondary dynamic recrystallization of the alloy is completed, and all small grains in the alloy grow into large secondary dynamic recrystallization grains. To ensure better grain refinement effect, the deformation temperature of the alloy should not be too high.

**Figure 7:**
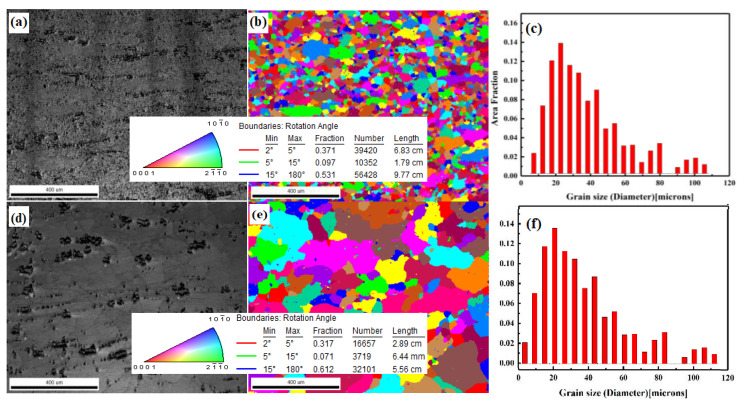
EBSD images of the alloy during hot compression at the strain rate of 0.001 s^−1^: (**a**) original image at 653 K; (**b**) orientation imaging at 653 K; (**c**) grain statistics at 653 K; (**d**) original image at 733 K; (**e**) orientation imaging at 733 K; (**f**) grain statistics at 733 K.

When the strain rate is constant (0.001), the grain size of the alloy sharply increases with an increase in temperature (Figure 7). It is shown that the grain size of the alloy is only 38 µm at 653 K and increases to 134 µm at 733 K. With an increase in temperature, the recrystallization process is accelerated, which leads to an increase in grain size.

### 4.2. Effect of Strain Rate on Microstructure

**Figure 8:**
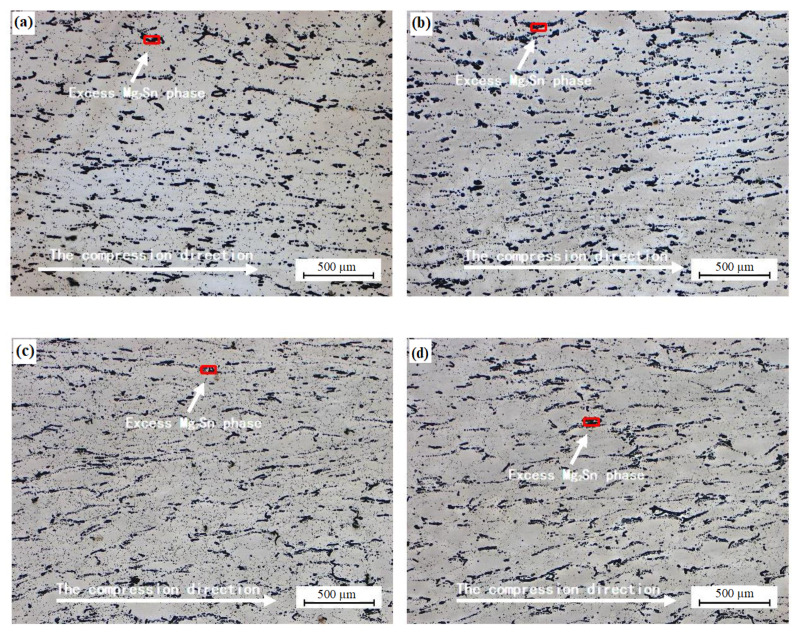
Microstructures of the alloy during hot compression at the temperature of 733 K. (**a**) 0.001 s^−1^; (**b**) 0.01 s^−1^; (**c**) 0.1 s^−1^; (**d**) 1 s^−1^.

**Figure 9:**
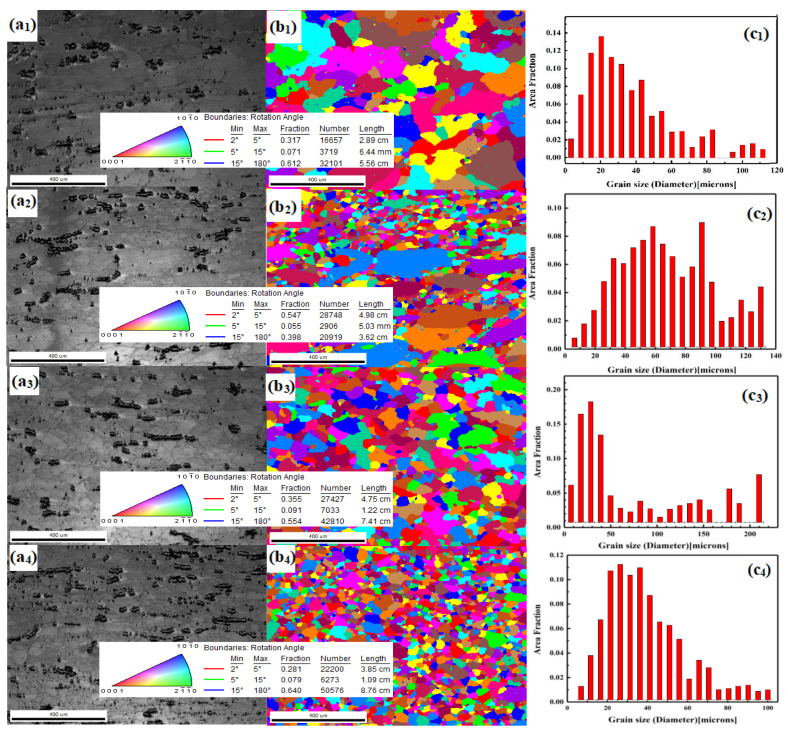
EBSD images of the alloy during hot compression at 733 K. (**a_1_**–**a_4_**) are the original images at 0.001, 0.01, 0.1, and 1 s^−1^, respectively; (**b_1_**–**b_4_**) orientation imaging at 0.001, 0.01, 0.1, and 1 s^−1^, respectively; (**c_1_**–**c_4_**) grain statistics at 0.001, 0.01, 0.1, and 1 s^−1^, respectively.

The recrystallized grain size of the Mg–8 wt.% Sn–1.5 wt.% Al alloy decreases with an increase in the strain rate when the strain rate is 0.001, 0.01, 0.1, and 1 s^−1^ at 733 K. The recrystallized grain size grows perpendicular to the direction of compression and along the tensile grain boundaries; the secondary phase shows a striped structure (Figure 8). The recrystallized grains nucleate near the grain boundary where the stress is strongest, and the dynamically recrystallized grains near the second phase are finer. When the temperature is constant, with a decrease in the strain rate, the recrystallization process has enough time to complete, which leads to secondary recrystallization and an increase in the grain size (Figure 9b_1_–b_3_). When the strain rate is 1, 0.1, 0.01, and 0.001 s^−1^, the grain size of the alloy is 19, 30, 62, and 76 µm, respectively (Figure 9c_1_–c_3_).

### 4.3. Texture Analysis under Different Deformation Conditions

**Figure 10:**
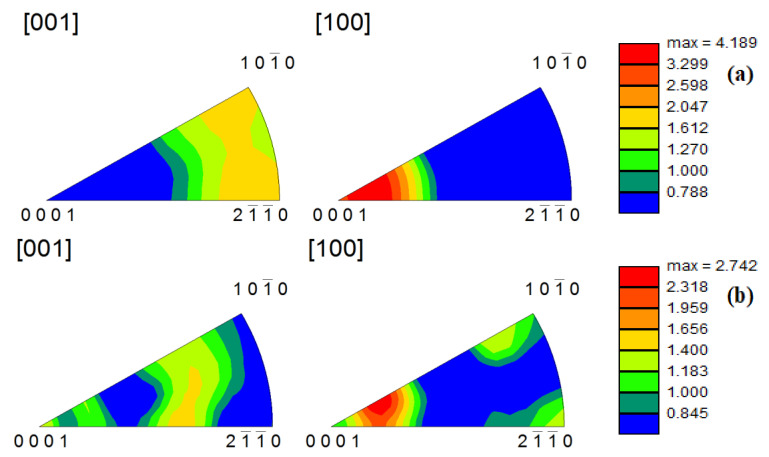
Anti-pole diagrams of the alloy during hot compression at 0.001 s^−1^ (**a**) 653 K; (**b**) 733 K.

**Figure 11:**
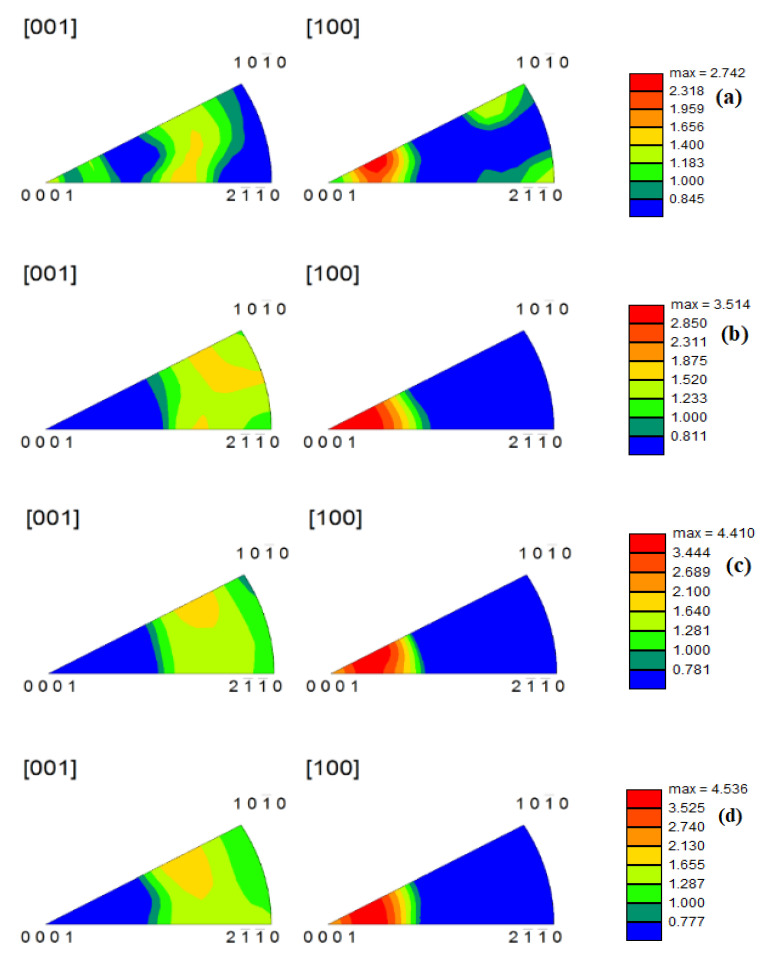
Anti-pole diagrams of the alloy during hot compression at 733 K (**a**) 0.001 s^−1^; (**b**) 0.01 s^−1^; (**c**) 0.1 s^−1^; (**d**) 1 s^−1^.

The c-axis of deformed grains was perpendicular to the compression direction at different temperatures. The textural strength decreased (Figure 10) (from 4.410 to 4.189) with an increase in temperature. However, an increase in texture strength is not clear because alloy recrystallization was completed at low temperature. Under compression at 633 K and at different strain rate, the alloy underwent complete recrystallization and already increased in size. The texture is along the basic (0001) direction, and the textural strength (Figure 11) increased (from 2.742 to 4.536) with an increase in strain rate. Under 0.001–s^−1^ deformation, the texture is not strictly along the basic (0001) direction; it is beneficial to reduce anisotropy by reducing the strain rate.

## 5. Conclusions

The hot deformation behavior of the Mg–8 wt.% Sn–1.5 wt.% Al alloy is studied by the hot compression test. Compared with other magnesium alloys, the processing map of the Mg–8 wt.% Sn–1.5 wt.% Al alloys also show certain instability and suitable zones for processing. However, the instability zones on the processing map of the alloy are small and the machinable zones are large. The deformation of the alloy also requires a certain deformation activation energy, which is 153.5 kJ/mol. The effects of the strain rate and deformation temperature on the flow stress of the alloy are discussed. The deformation activation energy, strain index, and hardening rate curve of the tested alloy are calculated; finally, the flow stress equation of plastic deformation is established. It is determined that the alloy has a wide hot working interval and can complete the thermoplastic deformation well. So, the alloy is easily deformed, which is important for its development and application.

The main conclusions are as follows:

(1) Steady-state rheological characteristics of the Mg–8 wt.% Sn–1.5 wt.% Al alloy exist in the process. The flow stress increases with an increase in the deformation degree and gradually decreases to a certain steady-state value after reaching a certain peak, which has clear dynamic recrystallization behavior characteristics. With a decrease in the deformation rate and an increase in the deformation temperature, the flow stress decreases. By comparing the influence of strain rate and deformation temperature on the flow stress curve, it is determined that the change in deformation temperature is more sensitive to the influence of the curve.

(2) During hot deformation, the stress, strain rate, and deformation temperature rheological relationship is expressed by the hyperbolic sine function, i.e., $A{\left[sinh\left(\alpha \sigma \right)\right]}^{n}=\overset{\cdot }{\varepsilon }exp\left(Q/RT\right)$; the deformation activation energy (*Q*) is 153.5 KJ/mol; the stress index (*n*) is 3.88; the average stress factor (*α*) is 0.034. The constitutive relationship (Equation (10)) of the alloy during hot compression deformation is calculated, which can verify and predict the machining properties of the alloy.

(3) By analyzing the microstructure of the Mg–8 wt.% Sn–1.5 wt.% Al alloy during deformation at different temperatures and strain rates, it is determined that the grain size of the alloy sharply increases (from 38 µm at 653 K to 134 µm at 733 K) with an increase in temperature, and the strength of the texture in the alloy decreases (from 4.410 to 4.189). At the same temperature, the grain size of the alloy decreases (from 76 µm at 0.001 s^−1^ to 19 µm at 1 s^−1^) with an increase in the strain rate, and the strength of the texture in the alloy decreases (from 4.536 to 2.742). Therefore, the increase of the deformation temperature will cause the growth of the grain size, as well as weaken the texture; on the contrary, the increase of deformation strain rate will reduce the grain size, as well as strengthen the texture.

## Figures and Tables

**Table 1:** Peak stress of the Mg–8 wt.% Sn–1.5 wt.% Al alloy.

Strain Rate	653 K	693 K	733 K	773 K
0.001 s^−1^	24.9 MPa	14.62 MPa	10.73 MPa	10.25 MPa
0.01 s^−1^	36.65 MPa	25.3 MPa	17.59 MPa	16.64 MPa
0.1 s^−1^	54.24 MPa	38.84 MPa	29.51 MPa	24.89 MPa
1 s^−1^	71.44 MPa	60.52 MPa	45.18 MPa	34.8 MPa

**Table 2:** 1/*n_1_* of the alloy at different temperatures.

Temperature	653 K	693 K	733 K	773 K	Average
1/*n*_1_	0.154	0.204	0.210	0.177	0.186

## Data Availability

The data presented in this study are available on request from the corresponding author.

## Nomenclature

R	the gas constant (8.314 J/K)
T	the Kelvin temperature
Z	the constant of hot deformation
*A*_1_	the material constants
*A*_2_	the material constants
*n*_1_	the material constants
*α*	the material constants
*β*	the material constants
*σ*	Stress
$\overset{\cdot }{\varepsilon }$	strain rate
*n*	Stress index
*P*	the energy absorbed per unit volume during hot deformation
*G*	energy of dissipation
*J*	energy related to the microstructure change during deformation
*m*	the strain rate–sensitive coefficient
*η*	dissipative power factor
*a*	temperature–dependent constants
*b*	temperature–dependent constants
*c*	temperature–dependent constants
*d*	temperature–dependent constants
